# A 0.73 dB Multi-Gain Low Noise Amplifier Design with Fast Mode-Switching for 5G/4G Applications

**DOI:** 10.3390/s24248082

**Published:** 2024-12-18

**Authors:** Kyung-Duk Choi, SungHwan Paik, Kyung-Jin Lee, Dong-Min Kim, Jun-Eun Park, Sang-Sun Yoo, Keum-Cheol Hwang, Youngoo Yang, Kang-Yoon Lee

**Affiliations:** 1 Department of Electrical and Computer Engineering, Sungkyunkwan University, Suwon 16419, Republic of Korea; glyiiop@skku.edu (K.-D.C.); kdm2357@skku.edu (D.-M.K.); juneun.park@skku.edu (J.-E.P.); khwang@skku.edu (K.-C.H.); yang09@skku.edu (Y.Y.); 2 SKAIChips, Suwon 16571, Republic of Korea; shback99@skaichips.co.kr (S.P.); kjsj1221@skaichips.co.kr (K.-J.L.); syoo@skaichips.co.kr (S.-S.Y.)

**Keywords:** RF LNA, gain mode switching, three-core input structure, cascaded switching, reconfigurable input structure

## Abstract

In this paper, a sub-1dB Low Noise Amplifier (LNA) with several gain modes, including amplification and attenuation modes required for the fifth and fourth generations (5G/4G) of mobile network applications, is proposed. Its current consumption is adaptive for every gain mode and varies to lower currents for lower amplifications due to the importance of current consumption for mobile network applications. The proposed LNA features an innovative architecture with a three-core input structure supporting multi-gain modes, achieving high gain and ultra-low noise performance. Additionally, the design integrates a cascade switching mechanism to ensure fast transitions between the gain modes and maintain operational stability. A reconfigurable input structure is introduced to support multiple input stages, enabling the proposed LNA to be compatible with both 5G and 4G applications. The proposed design demonstrates the implementation of seven distinct gain modes with a maximum current consumption of 11.68 mA, achieving proper input matching in each gain mode. The LNA delivers a maximum gain of 20.4 dB with a noise figure of 0.73 dB. Moreover, the most stringent mode switching condition achieved, the ON time, is as short as 1.295 µs, and the gain mode transition speed is an impressive 0.874 µs, ensuring extremely fast mode transitions. The proposed LNA occupies an area of 700 µm × 500 µm and is fabricated using a 65 nm FD-SOI process.

## 1. Introduction

The demand for high-speed data transmission has significantly increased in recent years, driven by the rapid evolution of mobile communication systems. In this context, the Low Noise Amplifier module has become a critical component, especially in extending cellular coverage and enhancing signal sensitivity [1]. Achieving the lowest possible noise figure (NF) is essential, as it directly impacts the receiver’s sensitivity, allowing for improved signal reception even at weak input levels. This is crucial in modern communication environments where maintaining strong connectivity across vast areas is paramount [2]. Additionally, a high-gain LNA is vital for amplifying weak signals without adding significant noise, thereby preserving the integrity of the received signal [3]. Furthermore, the dynamic nature of modern communication systems necessitates LNAs that can rapidly adapt to fluctuating signal conditions. Fast gain switching in LNAs provides substantial benefits in these environments, particularly where signal conditions can change abruptly due to factors like user movement or varying interference levels [4]. Quick gain transitions help maintain optimal signal quality, reducing the likelihood of dropped connections or data errors. In real-time applications, such as live streaming or video calls, the ability to swiftly adjust the gain ensures that the system can respond immediately to changing conditions, minimizing latency and preventing data loss.

Additionally, fast gain switching contributes to overall power efficiency, as the system can reduce gain when high gain is unnecessary, conserving power without compromising performance. Previous research, such as that presented in [5], explores the use of basic cascade topologies to achieve high gain. While this approach effectively increases gain, it does not address the need for versatile gain control across various modes. Achieving multiple gain levels requires more sophisticated design techniques that can manage the trade-offs between gain, noise, and other parasitic elements that could degrade overall performance. Therefore, designing an LNA capable of supporting multiple gain modes necessitates careful consideration of these factors to ensure that the LNA maintains high performance across all modes. In [6], a conventional approach to LNA design is discussed, where the focus is on achieving multiple gain modes. The proposed method also highlights the importance of ensuring high mode-switching speed and operational stability, which are crucial for reliable performance in real-world applications.

**Figure 1:**
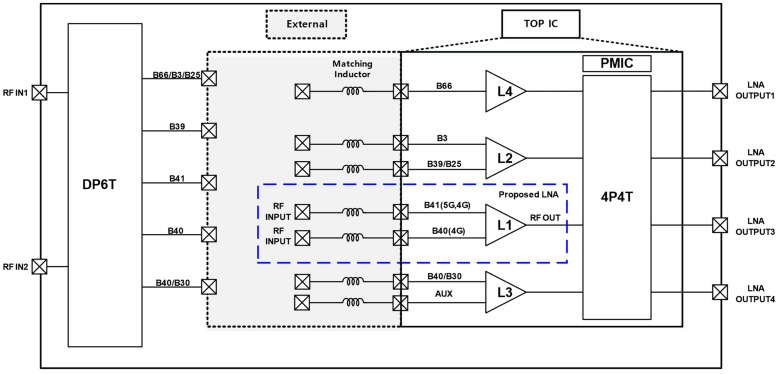
Block diagram of diverse RF front-end for 5G/4G Application.

The ability to rapidly switch between gain modes while maintaining stability ensures that the LNA can adapt to varying signal conditions without introducing instability or excessive noise. Figure 1 illustrates a block diagram of a diverse RF front-end suitable for multi-mode cellular handsets, highlighting the role of external inductors for input matching and the use of SAW filters for band filtering in each frequency band. Generally, a Low Noise Amplifier is designed with a single input port and a single output port structure [7]. This single-port configuration is effective in minimizing circuit complexity and ensuring stable amplification performance by reducing noise. However, when configured in a Multiple-Input Multiple-Output (MIMO) system, the LNA circuit becomes significantly more complex [8].

In a MIMO structure, multiple channels simultaneously receive and process signals, making it crucial to avoid interference between circuits. In this process, there is a high possibility of noise increase, and if adequate isolation performance between the bands is not ensured, signal interference can lead to degradation in overall noise and gain performance. Furthermore, if the switching speed is not sufficiently fast when changing between frequency bands, there may be instances of signal loss, resulting in a drop in communication quality. On the other hand, maintaining a single-port structure while handling multiple frequency bands would require independent LNAs for each band. This approach would lead to a drastic increase in chip size as the number of required LNAs increases with the number of frequency bands. From a practical standpoint, this not only hinders the commercial viability and feasibility of the product, but also significantly increases manufacturing costs and power consumption. Therefore, the conventional LNA structure is not suitable for efficiently handling the multiple bands demanded by modern communication systems.

In this paper, we propose an LNA that covers both B40 and B41 frequency bands to address these challenges. The proposed LNA effectively resolves issues such as circuit complexity and noise increase due to signal interference between bands in MIMO configurations while ensuring a sufficiently fast switching speed for band transitions. To achieve this, we introduce a cascaded switching mechanism technique that allows efficient handling of both frequency bands, maintaining noise performance and minimizing inter-band interference. This enables the proposed LNA to meet the performance and stability requirements of multi-band MIMO systems.

## 2. LNA Architecture and Core Design

**Figure 2:**
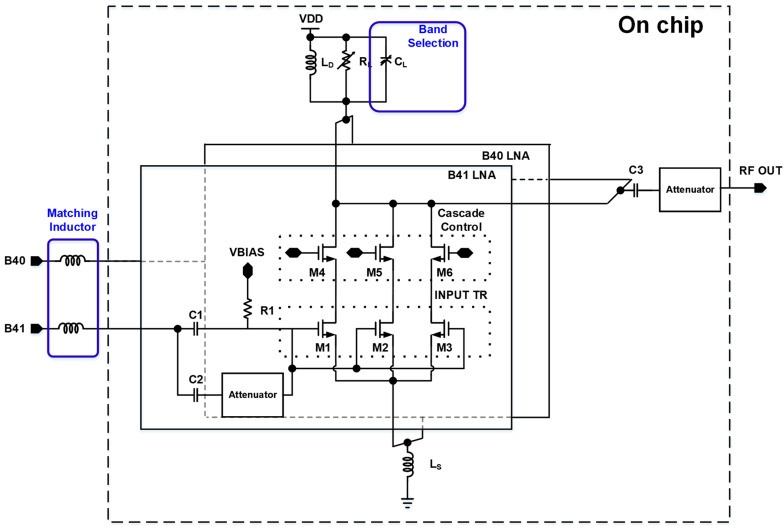
Proposed Low Noise Amplifier structure.

The proposed LNA structure uses conventional cascade configurations to achieve high gains, and obtains optimal noise figure using Custom NF Matching methods. Figure 2 illustrates the architecture of the proposed three-core LNA, designed to achieve seven distinct gain modes. This design employs three separate Common Source amplification stages, allowing the LNA to cover a wide range of gain values. The division of the amplification stages into three cores is a strategic choice that enables the system to adjust gain dynamically and efficiently across a broad spectrum. The proposed architecture introduces a novel approach to gain adjustment by keeping the bias voltage continuously applied to the cores, irrespective of whether they are in use. This constant biasing ensures that the gain mode transitions are swift, as it eliminates the need for additional charging time that would otherwise be required if each core’s bias were independently controlled. The ON/OFF switching of the cores is instead managed using a cascaded stage, which facilitates rapid transitions between gain modes. By maintaining a steady bias and controlling the core activation through cascaded ON/OFF logic, the design not only achieves fast gain mode switching but also ensures that there is minimal interaction between the input stages. This isolation between the inputs is crucial for maintaining operational stability, especially when the LNA operates across different gain modes. If each core’s bias were managed independently, the mode transition would be hindered by the RC delay associated with charging the core’s gate voltage, necessitating a settling time that would impede rapid transitions.

However, by employing cascaded ON/OFF switching logic, the proposed design circumvents this issue, achieving much faster switching speeds. The gain range achieved by this LNA spans from 20.3 dB to −12 dB, with noise figure performance varying between 0.73 dB and 15 dB. These performance metrics are critical for ensuring that the LNA can operate effectively across various signal environments, providing both high sensitivity and low noise when needed. To achieve multiple gain modes, it is essential to carefully design the input and output stages with a combination of resistors and capacitors that can control both the gain and the noise figure. In the proposed LNA, the key performance parameters—namely, maximum gain and minimum noise figure—are achieved by configuring the input and output stages in their purest form. The ability to control the noise figure is directly tied to minimizing the gain’s parasitic elements and optimizing the amplifier’s overall design. The ability to control noise figure performance would help to minimize the gain and parasitic components of the amplifier. The following shows the equation of the noise figure.

(1) $$NF\left(f\right)=10log(1+\frac{kT\left(G_{amp}\left(f\right)-1\right)+kTfC_{p}R_{s}}{N_{in}\left(f\right)})$$

The noise performance of a Radio Frequency Low Noise Amplifier (RF LNA) is a crucial parameter that determines the overall effectiveness of the amplifier in communication systems. The noise contributions can be analyzed through two primary terms: $kT\left(G_{amp}\left(f\right)-1\right)$ and $kTfC_{p}R_{s}$.

The first term, $kT\left(G_{amp}\left(f\right)-1\right)$, represents the increase in noise due to the amplifier’s gain. As the gain of the amplifier increases, this term indicates that the noise contribution also rises. However, this increase in noise does not necessarily degrade the noise figure of the amplifier. This is because the signal at the output is amplified alongside the noise, allowing the overall NF to remain stable or even improve, provided the gain is sufficiently high relative to the noise increase.

In contrast, the second term, $kTfC_{p}R_{s}$, highlights the noise added by parasitic capacitance ($C_{p}$) in the circuit. Unlike the noise contribution from gain, the noise introduced by parasitic capacitance becomes more significant as the operating frequency increases. This increase in noise due to parasitics directly impacts the NF, leading to degradation in performance at higher frequencies. Therefore, minimizing parasitic capacitance is a critical design consideration, particularly in the layout phase, to ensure the LNA maintains a low NF across its operational frequency range. Moreover, the design must also take into account the resistive components that appear in series with the input stage. Generally, reducing the series parasitic resistance lowers the noise contribution from this source. However, this reduction often leads to an increase in parasitic capacitance. Therefore, a careful balance must be struck between these two factors to achieve the minimum possible NF at the target operating frequency. This requires precise layout design, where the interplay between resistance and capacitance is optimized to reduce overall noise. An exception to this approach occurs in the context of noise matching. In some cases, it is beneficial to intentionally add parasitic capacitance between the gate and source of the input transistor. This deliberate addition of capacitance can help achieve the minimum NF by aligning the noise impedance with the source impedance. However, aside from this intentional addition, it is crucial to minimize all other parasitic elements in the design to prevent unwanted noise contributions.

The proposed Low Noise Amplifier features a configuration with multiple input stages converging into a single output. Therefore, even if parasitic components are optimized in the layout, it is inevitable to avoid the parasitic components that arise from the input structure. The proposed LNA overcomes this limitation by handling multiple input stages without configuring switches, thereby minimizing the unavoidable parasitic components caused by the structure.

**Figure 3:**
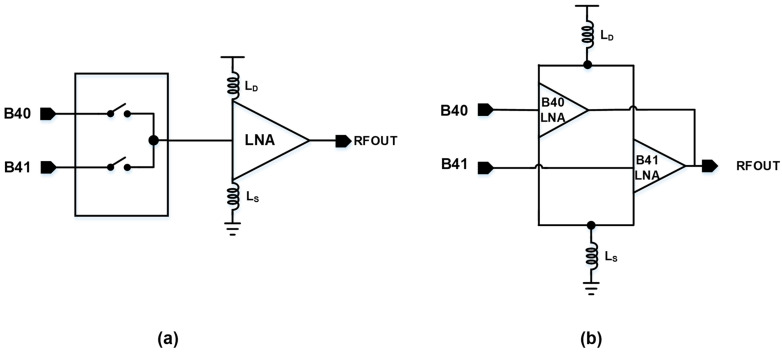
Methods for multi-input configuration: (**a**) general switch configuration; (**b**) multi-input reconfigurable LNA structure.

Typically, as illustrated in Figure 3a, such a setup would involve a switch array that matches the number of input stages to the LNA [9]. This conventional approach allows for the selection of different input stages depending on the signal requirements. However, a significant drawback of this conventional configuration is the linear degradation of noise figure performance with the increasing number of input stages. As the number of inputs grows, the additional switches introduce parasitic elements and insertion losses, which contribute directly to the overall NF degradation. The deterioration in the NF becomes more pronounced as the signal path lengthens due to the additional switching elements. The total noise, influenced by losses occurring at the switch, is defined by Equation (2).

(2) $$F_{switch}=\frac{S_{in}/N_{in}}{S_{out}/N_{out}}=\frac{S_{in}/kTB}{\frac{1}{L}S_{in}/kTB}=L_{switch}$$

where $S_{in}/N_{in}\;$ represents the signal-to-noise ratio (SNR) at the input, while $S_{out}/N_{out}$ represents the SNR at the output. When multiple input stages are employed, $S_{in}/N_{in}$ is directly influenced by the number of input stages. As the number of input stages increases, the physical size and complexity of the input network grow, leading to a linear increase in noise components. This increase in noise is a critical factor, as it directly affects the overall noise figure of the system. In the specific context of this paper, which deals with the 2.3 to 2.7 GHz frequency range across two bands, a Dual-Port Single-Throw (DPST) switch design is required. The DPST design allows the system to handle multiple frequency bands efficiently, but it also introduces a new challenge: the insertion loss of the switch. The insertion loss at this stage typically ranges between 0.2 and 0.3 dB. According to the Friis equation, as defined in Equation (3), this insertion loss is directly added as noise at the LNA input, further contributing to the NF.

(3) $$\begin{array}{c} F_{tot}=F_{switch}+\frac{F_{LNA}-1}{L^{-1}}=L+(F_{LNA}-1)L=L_{switch}\cdot F_{LNA}\\ NF_{tot}=L_{switch}+NF_{LNA}(\mathrm{in}\;\mathrm{dB}) \end{array}$$

This direct addition of noise due to the switch’s insertion loss highlights the trade-offs involved in multi-band LNA designs. While a DPST switch design enables the LNA to operate across multiple frequency bands, the accompanying noise penalty must be carefully managed to maintain a low NF. This consideration is especially important in applications requiring high sensitivity and low noise, where even small increases in noise can significantly impact the overall performance. In this paper, we propose a novel reconfigurable input structure, as illustrated in Figure 3b. When a signal is input to either the B41 or B40 input port, the M4, M5, and M6 elements of the unused input LNA are turned off to ensure isolation performance between ports. Unlike conventional designs that rely heavily on switches for input selection and signal routing, the proposed architecture eliminates the need for switches by introducing reconfigurable cores that share the load and degeneration inductors. Isolation performance between the output and the unused input is crucial, and the proposed architecture ensures sufficient performance of over 30 dB. This innovative approach allows for the creation of independent LNA channels for each input stage, thus maintaining the integrity of the signal path while improving overall performance.

One of the primary advantages of this design is the removal of switches at the input stage, which effectively eliminates the switch-related noise degradation that is typically unavoidable in traditional designs. By doing so, the design not only improves the noise figure but also simplifies the signal routing, reducing complexity. Channel isolation, which is usually achieved through switches, is instead realized through cascaded on/off control mechanisms, ensuring that each channel remains isolated without introducing additional noise. When operating in low-gain modes, the proposed structure adjusts the gain through attenuators placed at both the input and output stages. This design option allows the fine-tuning of gain in relatively high-gain modes, G0, G1, and G2, without compromising noise performance or signal integrity. Additionally, frequency matching is accomplished through carefully designed capacitance combinations at both the input and output, ensuring that the LNA remains optimally matched across a wide range of frequencies. The proposed reconfigurable input structure offers significant improvements in terms of noise performance, channel isolation, and flexibility, making it a robust solution for modern RF LNA applications.

## 3. Measurement Results

The proposed LNA employs a Flip Chip–Chip Scale Package (FC-CSP) to achieve optimal performance in high-frequency applications. This packaging choice is critical, as it directly influences the overall size, thermal management, and electrical performance of the LNA. One of the key features of the FC-CSP used in this design is the inclusion of a Sub-PCB, which plays a vital role in the input matching process. The Sub-PCB is specifically designed to mount the input matching inductor, a critical component that significantly affects both the gain and noise figure of the LNA. Due to the high sensitivity of the LNA to parasitic elements, the design of the Sub-PCB must carefully account for parasitic inductance and capacitance, which can otherwise degrade the LNA’s performance. In this context, the design of the Sub-PCB is not merely a passive aspect of the overall architecture, but is instead a crucial element that requires careful consideration. The layout must minimize parasitic effects to maintain the integrity of the input signal, ensuring that the LNA operates with optimal gain and low noise.

**Figure 4:**
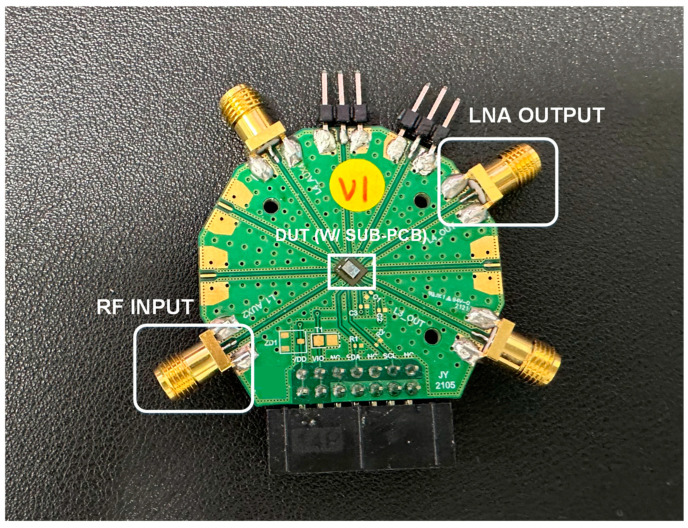
Board configuration for LNA measurement.

**Figure 5:**
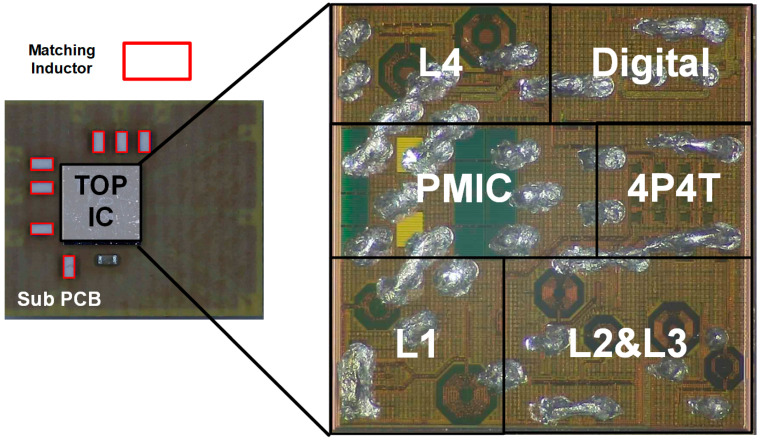
Die photo of proposed LNA IC.

This involves precise control over the physical placement and routing of components on the Sub-PCB to mitigate unwanted parasitic elements that could introduce additional noise or loss into the system. Figure 4 shows the measurement board. The proposed LNA’s S-parameter performance was comprehensively verified using a Network Analyzer (E5071C, Keysight Technologies, Santa Clara, CA, USA) as the measurement equipment. Additionally, the operational speed in the time domain, among other aspects, was visually confirmed using an RF Signal Generator (E4438C, Agilent Technologies, Santa Clara, CA, USA) and Oscilloscope (DSOX3024T, Keysight Technologies, Santa Rosa, CA, USA). Figure 5 presents a die photo of the module containing the proposed LNA. As depicted in the block diagram in Figure 1, the die photo includes four LNAs labeled L1 through L4. The module’s output can be routed to the desired antenna through four outputs using a 4P4T switch. This paper specifically focuses on the L1 LNA. Additionally, the module integrates a PMIC and digital circuitry. The Sub-PCB is designed to accommodate the module IC and the matching inductors.

**Figure 6:**
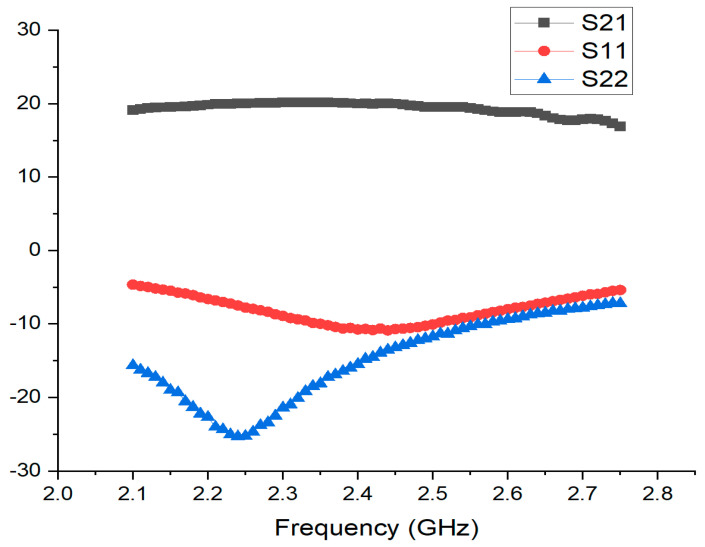
Results of measurement of gain and return loss in maximum gain mode of proposed LNA.

**Figure 7:**
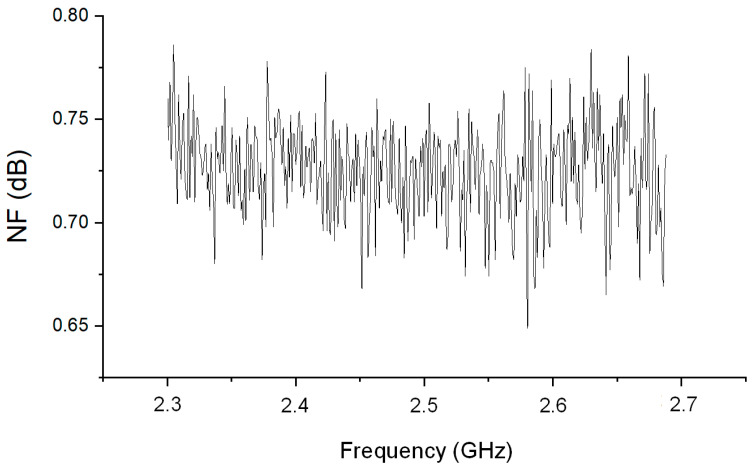
Measured noise figure at maximum gain of proposed LNA.

Figure 6 illustrates the input and output return loss values alongside the gain in Max Gain Mode. To evaluate the performance of the proposed LNA, measurements were taken across the target frequency range of 2.3 GHz to 2.7 GHz. The results demonstrate that the proposed LNA achieves a maximum gain of 20.4 dB in the Max Gain Mode. This high gain is consistently maintained across the entire frequency range, indicating robust performance even at higher frequencies. Notably, the return loss for both the input and output is observed to be consistently at least −8 dB throughout the frequency band. This suggests that the LNA provides excellent matching and signal transmission efficiency across the specified frequency range. These results highlight that the proposed LNA design is optimized to deliver high gain and superior return loss performance across a broad frequency range. This is a critical feature for RF communication systems, ensuring reliable performance and excellent signal quality in practical applications. Figure 7 presents the noise measurement results for the proposed LNA in Max Gain Mode. The noise performance is assessed across the frequency range of 2.3 GHz to 2.7 GHz, highlighting the efficiency of the LNA in maintaining low noise levels. The measurement results reveal that the LNA achieves a noise figure ranging from approximately 0.7 dB to 0.75 dB within the specified frequency band. This exceptional noise performance underscores the LNA’s capability to operate with minimal noise while delivering high gain. The consistency of the noise figure across the entire frequency range further demonstrates the effectiveness of the proposed design in ensuring superior signal quality and maintaining signal integrity. These results confirm that the proposed LNA design not only achieves high gain but also maintains a low noise figure, making it suitable for high-performance RF applications where minimizing noise is crucial for overall system performance. Table 1 presents the performance metrics corresponding to different gain modes. There are a total of seven modes, with G0 providing the highest gain and G6 the lowest. It can be observed that NF, P1dB, and current consumption vary according to the gain level in each mode.

**Figure 8:**
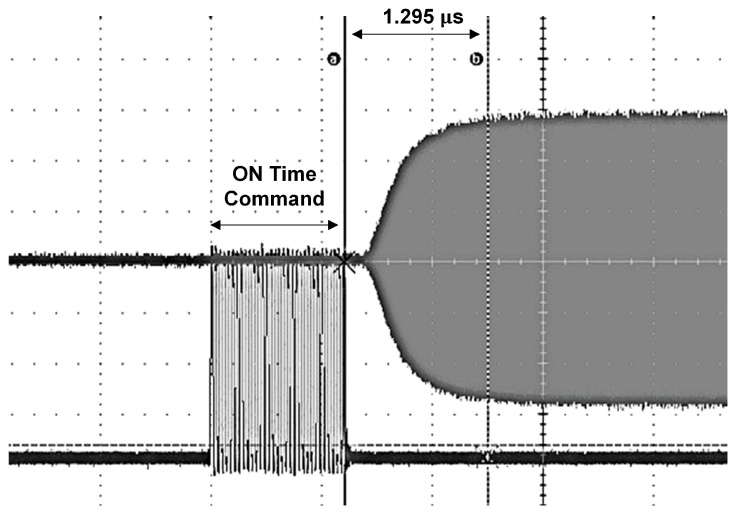
ON time measurement: waveform visualization.

Figure 8 depicts the ON time waveform for the proposed LNA. This waveform is crucial for evaluating the LNA’s switching performance, particularly under conditions when the LNA is initially non-operational. In this non-operational state, it is necessary to account for the time required to generate the bias voltage and for the core gate voltage to charge, which represents the most challenging scenario for LNA operation The measurement results reveal that the ON time, which represents the time required for the LNA to transition from a non-operational state to full operational status, is approximately 1.295 µs. This finding indicates that the LNA can achieve rapid switching performance even in the worst-case conditions. The ability to switch quickly is essential for applications requiring rapid response times and efficient mode transitions, ensuring that the LNA can promptly adapt to changing signal conditions while maintaining optimal performance. The demonstrated ON time of approximately 1.295 µs highlights the LNA’s capability for fast switching operations, making it suitable for high-speed RF applications where quick mode transitions and responsive signal acquisition are critical.

**Figure 9:**
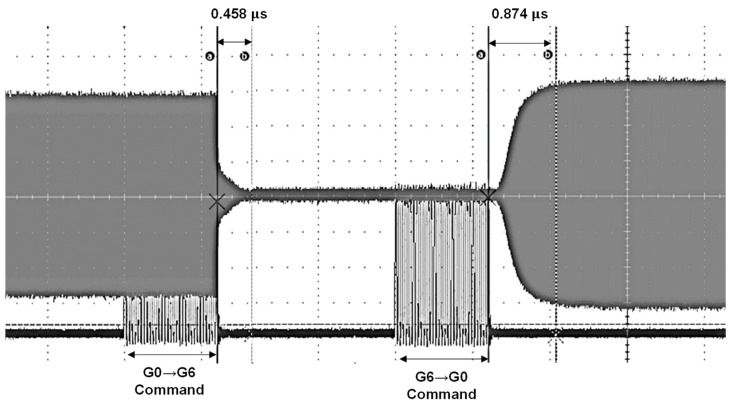
Mode switching time: waveform representation.

Figure 9 illustrates the mode switching waveform for the proposed LNA. In this analysis, G0 represents the highest gain mode, while G6 denotes the lowest gain mode. The waveform reveals that transitioning from the highest gain mode (G0) to the lowest gain mode (G6) involves the most significant number of factors being changed, which results in the longest switching time. This is due to the complex adjustments required when moving from a high-gain setting to a low-gain setting. The measured switching times demonstrate the LNA’s impressive performance: the transition from G0 to G6 takes approximately 0.458 µs, while the switch from G6 back to G0 is completed in about 0.874 µs. These results highlight the LNA’s capability for rapid mode switching, providing quick adaptation between different gain settings. Fast switching performance is essential for applications requiring swift changes in gain modes and optimal responsiveness to varying signal conditions. These results underscore the efficiency of the proposed LNA design in achieving rapid mode transitions, enhancing its suitability for high-speed RF applications where swift adaptation to different operational states is critical.

Table 2 presents the performance comparison of the proposed LNA design with other state-of-the-art designs. The comparison includes key performance metrics such as frequency range, gain, return loss, noise figure, and power consumption, which are essential indicators for evaluating the overall efficiency and effectiveness of an LNA. One of the most significant aspects highlighted in this table is the Figure of Merit (FoM). The FoM is calculated by dividing the gain by the product of the noise figure and power consumption. A higher FoM value indicates superior performance, as it reflects the LNA’s ability to achieve high gain while maintaining low noise and power consumption. The proposed design demonstrates a competitive and impressive FoM, signifying its efficiency in meeting the demanding requirements of modern communication systems. This emphasizes the effectiveness and robustness of our LNA architecture compared to other existing solutions.

## 4. Conclusions

In conclusion, the proposed Low Noise Amplifier leveraging the 65 nm FD-SOI process achieved exceptional noise figure and gain performance through an innovative design approach. Beyond the chip-level design, extensive efforts were made to minimize performance degradation caused by unintended parasitic components, with particular attention paid to the design of the PCB and Sub-PCB. The measurement results accounted for losses introduced by the Sub-PCB, and the degradation observed in the PCB lines was accurately compensated using de-embedding techniques. These results strongly indicate the effectiveness of our design methodology in enhancing LNA performance.

## Figures and Tables

**Table 1:** Performance by gain mode (@ 2.3~2.69 GHz).

Mode	Gain (dB)	NF (dB)	P1dB (dBm)	Current (mA)
G0	20.4/20.16	0.73/0.77	−20.46/−20.49	11.68/11.6
G1	18.3/17.9	0.77/0.79	−18.60/−18.56	11.6/11.6
G2	12.12/12	0.94/0.95	−13.21/−13.5	7.15/7.1
G3	6.8/6.9	2.2/2.2	−12.49/−12.8	5.9/5.5
G4	1.5/1.5	4.2/4.1	−8.9/−9	2.7/2.67
G5	−5.3/−5	9.2/9.1	−3.49/−3.13	0.97/0.88
G6	−10.5/−10.06	12.33/12.3	−1.9/−2	0.97/0.88

A/B format represents data corresponding to input B40 and input B41, respectively.

**Table 2:** Performance summary.

	[10]	[11]	[12]	[13]	[14]	This Work
Freq. (GHz)	5.9	0.1–3.4	0.02–4.5	4–11.5	0.3–3.5	2.3–2.7
Power Gain (dB)	9	18.2	11.2–20.4	21	14.6	20.4@G0
S11 (dB)	−11	N/A	N/A	−10	−10	<−8
NF (dB)	1.34	3.4	3.2–5.4	2.75	2.9	0.73@G0
IIP3 (dBm)	N/A	−1.46	−8	6.5	1.2	−10.5@G0
Power (mW)	9.6	3.3	15.6	5.15	14.8	11.68@G0
Tech. (nm)	130	130	28	65	180	65
On Time (µs)	-	-	-	-	-	1.295
Mode Switching Time (µs)	-	-	-	-	-	0.874@G6- > G0
FoM	0.6	9.15	3.36	12.97	1	7.94

$FoM=\frac{\left|S21\right|}{F\;*\;Power}.$

## Data Availability

The original contributions presented in the study are included in the article, further inquiries can be directed to the corresponding author.
